# Nanoemulsions of red quinoa and ginseng extracts with chitosan wall: Investigating the antioxidant properties and its effect on the shelf life of dairy cream

**DOI:** 10.1002/fsn3.4182

**Published:** 2024-05-21

**Authors:** Hadis Aziminezhad, Reza Esmaeilzadeh Kenari, Zeynab Raftani Amiri

**Affiliations:** ^1^ Department of Food Science and Technology Sari Agricultural Sciences and Natural Resources University Sari Iran

**Keywords:** chitosan, dairy cream, ginseng, oxidation stability, quinoa, shelf life

## Abstract

The study aimed to investigate the antioxidant properties of ginseng and red quinoa extract nanoemulsion and its effect on the shelf life of dairy cream. Nanoemulsion includes dairy cream, Tween 80, chitosan, whey protein powder, chitosan/whey protein powder, red quinoa extract, ginseng extract, and a mixture of extracts (1:1). The highest total phenol content and total flavonoid content were related to ginseng extract (24,009.55 mg of gallic acid equivalent/kg, 883.16 mg quercetin/kg) with ethanol–water solvent (80:20). Most of the phenolic and flavonoid compounds of ginseng and red quinoa extracts were related to p‐coumaric acid (211.3 μg/g), catechin (29.6 μg/g), ellagic acid (73.88 μg/g), and rutin (34.12 μg/g), respectively. Considerable antioxidant power in the concentration of 800 ppm of red quinoa and ginseng extracts (ethanol–water solvent (50:50), (80:20)) in 2,2‐diphenyl‐1‐picrylhydrazyl radical scavenging (80%, 82%, 80%, and 78%), bleaching β‐carotene: linoleic acid (81%, 73%, 77%, and 86%), and ferric reducing antioxidant power assays (70%, 73%, 72%, and 76%) was observed. Nanoemulsions of red quinoa extract with chitosan wall had the smallest particle size (250.67 nm), the highest encapsulation efficiency (72.79%), and the polydispersity index (0.34). Nanoemulsions containing ginseng + quinoa (1:1) with chitosan/whey protein powder wall showed the highest viscosity (5.30 mPa/s) and the mostzeta potential (−32.6 mv). Also, nanoemulsions of red quinoa extract showed the lowest amount of peroxide value and the thiobarbituric acid value (12 milliequivalent O_2_/kg–0.48 μg/mL) in dairy cream oil. In general, the red quinoa extract with chitosan wall was superior to other samples due to the delay in oxidation and positive effect on the shelf life of dairy cream.

## Graphical abstract

Ginseng extract had the highest total phenol content and total flavonoid content and most of its phenolic and flavonoid compounds were related to p‐coumaric acid and catechin. The concentration (800 ppm) of red quinoa extract (ethanol–water solvent (80:20)) in 2,2‐diphenyl‐1‐picrylhydrazyl radical scavenging, bleaching β‐carotene: linoleic acid, and ferric reducing antioxidant power assays were observed. The red quinoa extract nanoemulsions with chitosan wall had the smallest particle size, highest encapsulation efficiency, highest polydispersity index, and the lowest peroxide value and thiobarbituric acid value in dairy cream oil.

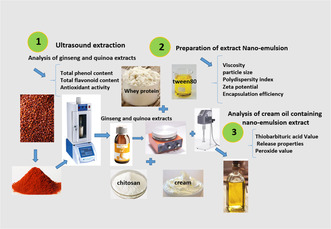

## 1 INTRODUCTION

Dairy cream is one of the fat‐based dairy products obtained from the physical separation of milk. It contains 10%–50% fat and 2% protein. It is used for cream, cheese, butter, and whey protein (Hussain et al., 2017). The most significant problem with lipids is their destruction by oxidation. Dairy products, especially dairy cream due to the high amount of fat (10%–50%), may undergo oxidation due to various factors (light, temperature, and air). Oxidation leads to bad taste, reduction of nutritional value, and even production of toxic and carcinogenic compounds in it and other food products. For several years, food manufacturers have been using synthetic antioxidants to prevent and delay the oxidation reaction in foods containing high fat and oils. On the other hand, there are serious concerns about the carcinogenic potential of synthetic antioxidants and being dangerous for human health (Arabshahi‐Delouee & Urooj, 2007). Essential oils and extracts containing bioactive compounds with antioxidant properties can replace synthetic antioxidants. In addition, essential oils and extracts are recognized as safe substances (Generally Recognized As Safe) that have few side effects on human health (Rodino & Butu, 2019; Tabee et al., 2008).

The ginseng plant (*Panax ginseng*) belongs to the *Araliaceae* family and grows throughout East Asia and Russia. Most studies have mentioned ginsenosides as the main bioactive component of ginseng (Davis & Behm, 2019). Phenolic compounds and various phenolic acids have also been found in ginseng products (Lee et al., 2015). The quinoa plant (*Chenopodium quinoa* Willd.) belongs to the *Chenopodiaceae* family, which is rich in macronutrient compounds (protein, polysaccharides, and fats) and micronutrients (polyphenols). Polyphenols (phenolic acids, flavonoids, and tannins) have antimicrobial and antioxidant effects (Park et al., 2017).

Research has shown that when extracts are added to the food matrix, they may interact with food compounds and change the bioavailability of phenolic compounds and the availability of nutrients (Silva et al., 2018). As a result, it is necessary to encapsulate bioactive compounds. In encapsulation, fine particles are coated as a core inside a wall of materials. Encapsulation of bioactive compounds protects them from harmful environmental effects and controls their release at the target site. The first step in encapsulation is the selection of a suitable wall, which is effective on the encapsulation efficiency (EE) and the release rate of the effective compounds of the extract (Vijeth et al., 2019). Encapsulation of bioactive compounds is done with various materials, including chitosan and whey protein. Chitosan is a cationic polysaccharide composed of D‐glucosamine and *N*‐acetyl‐D‐glucosamine units linked by O‐glycosidic β (1 → 4) linkages (Tavares & Noreña, 2019). Features, such as nontoxic, nonallergenic, biodegradable, antimicrobial activity, antioxidant activity, high water absorption, excellent properties of the gel, and film formation, have made chitosan a polymer with many uses in various pharmaceutical and food industries (Kenari et al., 2020). The whey protein isolate is a protein product available in the market, which has a high nutritional value and has the exclusive ability to form emulsions, gels, and foams, and is widely used in food products (Firebaugh & Daubert, 2005). So far, no research has been done on adding nanoencapsulated extracts of quinoa and ginseng to dairy cream. Therefore, the purpose of this study was to investigate the characteristics of ginseng and red quinoa extracts, and nanoencapsulated extracts (as a natural antioxidant compound) were used to increase their shelf life, and their oxidative stability was investigated.

## 2 MATERIALS AND METHODS

### 2.1 Materials

Dairy cream (fat 36%) was obtained from the Kale Amol dairy factory (Amol, Iran), and ginseng extract was obtained from Adonis Gol Daru Company (Tehran, Iran). Other chemicals used were purchased from Merck, Germany.

### 2.2 Methods

#### 2.2.1 Ultrasound‐assisted extraction of quinoa seed

The quinoa seed was dried (dry and dark place: temperature 25°C). Then it was ground into powder. It was passed through a sieve with 80 mesh. Quinoa powder was stored in a double‐layered dark polyethylene bag (4°C). Extraction was done by the method of Arlene et al. (2015) with a slight modification. The quinoa seed powder was added to the desired solvent (1:10) and ethanol–water solvent with ratios of (50:50) and (80:20) was placed separately under the ultrasonic waves of a probe‐type ultrasound (Movel Scientific Instrument Co., Ltd, China) (frequency of 37 Hz: intensity of 100: 40°C: 15 min). The upper part (of the extract) was separated after centrifugation (6814 g: 30 min). The solvent was removed by a rotary vacuum evaporator (Optima, Korea) (4 g/min: 40°C) (Arlene et al., 2015). The extract was dried (50°C) (freeze‐dryer, Zirbus, Germany) and turned into powder. The resulting phenolic powders were stored in a freezer (−18°C) in airtight and moisture‐proof containers until use (Chang et al., 2006).

#### 2.2.2 Total phenol content and total flavonoid content in ginseng and quinoa extracts

Total phenol content (TPC) in the ginseng and quinoa extracts was measured by the Folin–Ciocalteu method according to the research of Acquadro et al. (2020). A quantity of the extract (200 μL) (ginseng and quinoa) was combined with pure water (800 μL). After 6 min, 200 μL of Folin–Ciocalteu identifier was added. After adding sodium carbonate 1 N (2 mL), the mixture was incubated (1 h: 25°C). The absorbance was read (λ = 765 nm) (ultraviolet (UV)/Visible (Vis) spectrophotometer, Laxco Alpha‐1502, USA). Then the standard curve for this test was drawn with gallic acid equivalent (Acquadro et al., 2020). Also, the total flavonoid content (TFC) of ginseng and quinoa extracts was determined (Shraim et al., 2021). The TFC of extracts (ginseng and quinoa) was determined by aluminum chloride colorimetric method. Sodium nitrate (1.5 mL: 5%) was mixed with the sample (10 mL), then after 5 min of rest, sodium chloride (10%: 3 mL) and pure water (10.5 mL) were combined with the mixture. And finally, the vortex was done (10 s). The absorbance was read (λ = 510 nm). Then the standard curve for this test was drawn with quercetin. The TFC in the ginseng and quinoa extracts was expressed as mg of quercetin/kg of dry extract.

#### 2.2.3 Determination of phenolic and flavonoid compounds of ginseng and quinoa extracts

The gas chromatography–mass spectrometry (GC–MS) (Agilent, USA) was used to identify the components of the extract. It was equipped with an Rtx‐5MS column. The column length, inner diameter, and the stationary phase thickness were 30 m, 0.25 mm, and 0.25 μm, respectively.

The initial temperature, final temperature, rate of temperature increase, and the remaining temperature were 60, 290°C, 10°C/min, and 13 min, respectively. The carrier gas was helium. The relative percentage of each compound was obtained, according to the area under its curve in the GC chromatogram (Proestos et al., 2006).

#### 2.2.4 2,2‐Diphenyl‐1‐picrylhydrazyl **(** DPPH) radical scavenging

In this experiment, the stable DPPH radical compound was used as a reagent, and 100 μL of ginseng and quinoa extracts with different concentrations was added to 10 mL of 0.004% DPPH. After 30 min of heating (27°C), the optical absorption of the ginseng and quinoa extracts was read (λ = 517 nm). The synthetic antioxidant tert‑butylhydroquinone (TBHQ) at a concentration of 100 μg/mL was used as a positive control (Saviz et al., 2015).

#### 2.2.5 β‐Carotene: linoleic acid bleaching assay

In this test, the β‐carotene–linoleic acid solution was prepared. β‐Carotene (0.5 mg) was dissolved in 1 mL of chloroform. Then 50 μL of linoleic acid and 400 mg of Tween 40 were added to it and then mixed thoroughly. After that, chloroform was separated by the vacuum evaporation method. The oxygen‐saturated distilled water (200 mL) (30 min under 100 mL/min) was added with vigorous shaking. The β‐carotene–linoleic acid solution (2.5 mL) was transferred to the test tube. Then 350 μL of different concentrations of ginseng and quinoa extracts was added to the test tube after 48 h of heating. In incubation at a temperature of 27°C, the optical absorption of the samples was recorded at 490 nm. The amount of antioxidant activity was measured by comparing the light absorption of the samples with zero time and by the stability of the β‐carotene color. The synthetic antioxidant TBHQ (100 μg/mL) was used as a positive control (Saviz et al., 2015).

#### 2.2.6 Ferric reducing antioxidant power (FRAP) assays

To measure FRAP, acetate buffer solution (300 mM: pH = 3.6), iron chloride hexahydrate (FeCl_3_·6H_2_O) solution (20 mM), and 1 mM 2,4,6‐Tris(2‐pyridyl)‐s‐triazine (TPTZ) in 40 mM hydrochloric acid (HCL) solution were prepared. All solutions were mixed with a ratio of 10:1:1 (v/v/v). Then different concentrations (200, 400, 600, 800, and 1000 μg/mL) of ethanol–water (50:50) and (80:20) ginseng and quinoa extracts were dissolved with 300 μL of FRAP solution. It was incubated at 37°C. After 8 min, the absorbance of the samples was read by a spectrophotometer (λ = 593 nm). The reducing activity of different concentrations (200, 400, 600, 800, and 1000 μg/mL) ethanol–water (50:50) and (80:20) ginseng and quinoa extracts was calculated in terms of micromole (μmol) of ferric per milligram (mg). The synthetic antioxidant TBHQ (100 μg/mL) was used as a positive control (Agregán et al., 2017).

#### 2.2.7 Preparation of nanoemulsion

To prepare the nanoemulsion, first, the wall solutions were prepared with chitosan, whey protein, and chitosan/whey protein in equal proportions and were stirred well with a magnet in distilled water for 24 h before preparing the emulsion. Then the primary emulsions were prepared by mixing ginseng extract, quinoa extract, and ginseng + quinoa extract mixture (1:1) with wall materials in a ratio of 1:3. Then, the primary emulsion formed was mixed with dairy cream and Tween 80 emulsifier (1% of the weight of the dairy cream) in a ratio of 1:2. It was homogenized with a high‐speed homogenizer (Ultrathorax D‐500, Dragonlab, Malaysia) at a speed of 36,288 g for 8 min. Finally, to further reduce the size of nanoemulsion particles, a probe‐type ultrasound device was used with the maximum intensity of the device at a temperature of 37°C for 3 min (Hashtjin & Abbasi, 2015). For drying, a freeze‐dryer was used for 48 h at a temperature of −57°C and a pressure of 1.7 × 10^−5^ Pa (Chranioti et al., 2015).

#### 2.2.8 Measurement of apparent viscosity

The apparent viscosity of ginseng extract and red quinoa nanoencapsulated with different walls (chitosan, whey protein, and the combination of these two walls) was measured in millipascal second (mPa.s) by a Brookfield rotational viscometer (Brookfield, USA) with a CPA‐40Z conical spindle (Demisli et al., 2020).

#### 2.2.9 Measurement of particle size, polydispersity index, and zeta potential

Particle size, polydispersity index (PDI), and zeta potential were measured by dynamic light scattering (Malvern Zetasizer Nano ZS model, Malvern, UK) based on the laser light diffraction method at a temperature of 25°C (Amiri et al., 2021).

#### 2.2.10 EE of nanoencapsulates

The method of Jivan et al. (2014) was used to determine EE. First, 20 mL of alkaline water (pH = 10.5) was mixed with 0.6 g of nanoencapsulated extract powder and stirred for 30 min with a magnetic stirrer. Then it was centrifuged (1792 *g*: 10 min) to separate the aqueous phase from the solvent. Next, the upper phase was neutralized with concentrated acid (pH = 7). The amount of phenol present in the supernatant phase was determined by the Folin–Ciocalteu method. EE was calculated.

$$\mathrm{EE}\left(\%\right)=\frac{\mathrm{EP}}{\mathrm{PP}}\times 100$$

EP = Amount of encapsulated phenolic compounds; PP = The initial amount of phenolic compounds present (Jivan et al., 2014).

#### 2.2.11 Scanning electron microscopy (SEM) image of nanoencapsulates

Imaging of the surface morphology of prepared nanoencapsulates was done by an electron microscope (Kenari et al., 2020).

#### 2.2.12 Preparation of dairy cream treatments with nanoencapsulated extracts and tert‐butylhydroquinone (TBHQ) antioxidant and oil extraction

The best wall was determined according to the results obtained from the previous tests. Then ethanol–water (80:20) of ginseng, quinoa, and ginseng + quinoa extracts (800 μg/mL) nanoencapsulated extracts in that wall were added to the pasteurized dairy cream. The treatment containing TBHQ was also prepared (100 μg/mL). Oil was removed from dairy cream (14 days: 4°C). To prepare oil from cream, dairy cream (100 g) was mixed with 400 mL of ethanol and was shaken under the hood for 5 h. After the passage of 24 h, the suspension became two‐phase, which was separated by passing through a strainer. To remove the solvent from the dairy cream oil, it was placed in an oven (45°C) for a week. Then the dairy cream oil was kept in a tank (−18°C).

#### 2.2.13 Oil oxidation

##### Peroxide value and thiobarbituric acid (TBA) value

Peroxide value (PV) was calculated in treatments containing nanoencapsulated extracts and artificial antioxidant TBHQ for 14 days (Deepika et al., 2014). The TBA value was also performed after 14 days (Qiu et al., 2021).

##### Release properties of phenolic compounds

The release of phenolic compounds of extracts (quinoa and ginseng) was done in cream oil. As a result, first the oil was separated from the cream. Then, the amount of released phenol was determined by the following method. Oil and hexane were mixed in the same ratio. After vortexing, 2.5 mL of methanol–water solvent was added to it. Then it was vortexed again for 1 min. Then the solutions were centrifuged for 5 min at 2800 *g*. The oil phase was removed with a sampler, and the aqueous phase was kept. Finally, the aqueous phases of each sample were transferred to a 50 mL volumetric flask. The TPC was determined according to the Folin–Ciocalteu method. Absorbance at 765 nm wavelength was read by a spectrophotometer. The TPC was expressed based on milligrams (mg) of gallic acid equivalent/kg of extract (Acquadro et al., 2020; Vajić et al., 2015).

### 2.3 Statistical analysis

In this research, all the tests were done with three repetitions. SAS 9.4 M6 software (completely random design format) was used to analyze the data. Duncan's test was used to compare the means at the 5% probability level.

## 3 RESULTS AND DISCUSSION

### 3.1 Determination of phenolic and flavonoid compounds of ginseng and quinoa extracts

The extract's chemical component was analyzed through GC–MS analysis. The highest phenolic compounds in ginseng and quinoa extracts were related to p‐coumaric acid and ellagic acid, respectively, at the rates of 211.3 and 73.88 μg/g, dry weight basis (Tables 1 and 2). The highest amounts of flavonoid compounds in ginseng and quinoa extracts belonged to catechin and rutin, 29.6 and 34.12 μg/g, dry weight basis, respectively (Tables 1 and 2). GC–MS was used to further produce purified ginseng extracts. These compounds inhibited the oxidative activities of radicals and ended the free radical chain reaction (Mansor et al., 2022). Identification of these bioactive compounds will determine not only nutritional value but also potential health benefits (Pandya et al., 2023).

**TABLE 1:** Phenolic and flavonoid compounds of ginseng extract.

Phenolic compounds		Flavonoid compounds	
Compound	Content (μg/g, dry weight base)	Compound	Content (μg/g, dry weight base)
Protocatechuic acid	21.67	Naringin	7.23
Gentisic acid	15.22	Catechin	29.6
Syringic acid	89.34	Hesperetin	4.09
Phenolic chlorogenic acid	7.11	Rutin	3.41
p‐Coumaric acid	211.3	Myricetin	1.9
Ferulic acid	9.86	Quercetin	2.22
m‐Coumaric acid	111.09	Biochanin A	2.18

**TABLE 2:** Phenolic and flavonoid compounds of quinoa extract.

Phenolic compounds		Flavonoid compounds	
Compound	Content (μg/g, dry weight base)	Compound	Content (μg/g, dry weight base)
Vanillic acid	1.22	Naringin	2.1
Salicyclic acid	15.92	Rutin	34.12
Cinnamic acid	2.77	Myricetin	2.3
p‐Hydroxybenzoic acid	8.23	Quercetin	6.72
Syringic acid	9.22	Kaempferol	1.89
Caffeic acid	10.9	–	–
p‐Coumaric acid	8.71	–	–
Ferulic acid	22.41	–	–
Ellagic acid	73.88	–	–
Benzoic acid	52.89	–	–

### 3.2 TPC and TFC in ginseng and quinoa extracts

Ethanol–water extracts (50:50) of ginseng and quinoa showed no significant difference in TPC (Table 3). But other extracts had showed significant differences in TPC. Also, the highest TPC was observed in the ethanol–water extract (80:20) of ginseng (24,009.55 mg of gallic acid equivalent/kg). When the TPC is higher, the number of hydroxyl groups in the reaction medium is higher. As a result, hydrogen donation to free radicals increases, and the antioxidant power of the extract increases (Wang et al., 2014). The TPC in the ginseng extract in this research was higher than that of Kim et al. (2007). The difference may be related to genetic and environmental conditions that affect the presence of phenolic compounds (Sekhavatizadeh et al., 2021). The results showed that there was a statistically significant difference between TFC of ginseng and quinoa extracts (Table 4). Also, TFC in the ethanol–water extracts (80:20) of ginseng and quinoa was higher than in the ethanol–water extracts (50:50) of ginseng and quinoa extracts, respectively. Ethanol–water extract (80:20) of ginseng with 30.21 mg of quercetin per kg of extract had the highest TFC. The TFC of ginseng and quinoa extracts in this research was consistent with the research of Malathy et al. (2020) and Buitrago et al. (2019), respectively. The total phenolic content in quinoa seeds with different genotypes studied by Pandya et al. (2023) changed from 35.51 mg/100 g to 93.23 mg/100 g dry weight of the seed (Pandya et al., 2023). Melini and Melini (2021) reported that they extracted the amount of phenolic compounds from black quinoa with ultrasound (aqueous ethanol solvent), which is consistent with the results of this research (Melini & Melini, 2021). Mansor et al. (2022) reported that the amount of phenolic compounds in ginseng root was more than twice its flavonoid compounds, which is consistent with the results of this study (Mansor et al., 2022). As one of the strongest antioxidants in plants, flavonoids prevent lipid peroxidation and act as scavengers of radicals, such as superoxides, peroxyl lipids, and hydroxyls (Yuting et al., 1990). Research has shown that the type of solvent is very effective in extracting phenolic and flavonoid compounds. So, the solubility of phenolic and flavonoid compounds in water is very low. When water is used to extract these compounds, the polysaccharides and proteins harm the extraction. They minimize the amount of extraction. In this research, a mixture of water and ethanol with different proportions was used. Because it has been proven that the mixture of water and ethanol increases the extraction of compounds from polysaccharide matrices (Maqsood et al., 2015).

**TABLE 3:** Comparison of phenolic composition of ginseng and quinoa extracts.

Type of extract	Amount of phenolic compounds (mg of gallic acid equivalent/kg)
Ethanol–water extract (80:20) of ginseng	24,009.55 ± 375.05^a^
Ethanol–water extract (50:50) of ginseng	21,493.86 ± 445.10^c^
Ethanol–water extract (80:20) of quinoa	22,710 ± 594.22^b^
Ethanol–water extract (50:50) of quinoa	21,324.4 ± 265.67^c^

*Note*: Different letters in a column indicate significant differences (*p* < .05).

**TABLE 4:** Comparison of flavonoid composition of ginseng and quinoa extracts.

Type of extract	Amount of flavonoid compounds (mg quercetin/kg)
Ethanol–water extract (80:20) of ginseng	883.16 ± 30.21^a^
Ethanol–water extract (50:50) of ginseng	755.30 ± 23.29^b^
Ethanol–water extract (80:20) of quinoa	580.89 ± 24.93^c^
Ethanol–water extract (50:50) of quinoa	533.7 ± 19.50^d^

*Note*: Different letters in a column indicate significant differences (*p* < .05).

### 3.3 Antioxidant activity of ginseng and quinoa extracts

#### 3.3.1 2,2‐Diphenyl‐1‐picrylhydrazyl (DPPH) radical scavenging

**FIGURE 1:**
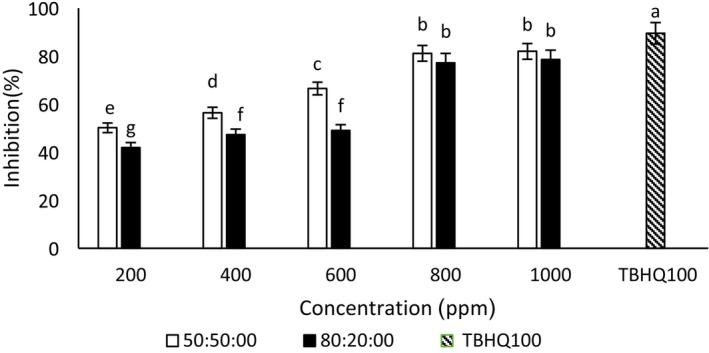
2,2‐diphenyl‐1‐picrylhydrazyl (DPPH) radical inhibition of different concentrations of ginseng–ethanol‐water extract (50:50) and (80:20). Different letters indicate significant differences (*p* < .05).

**FIGURE 2:**
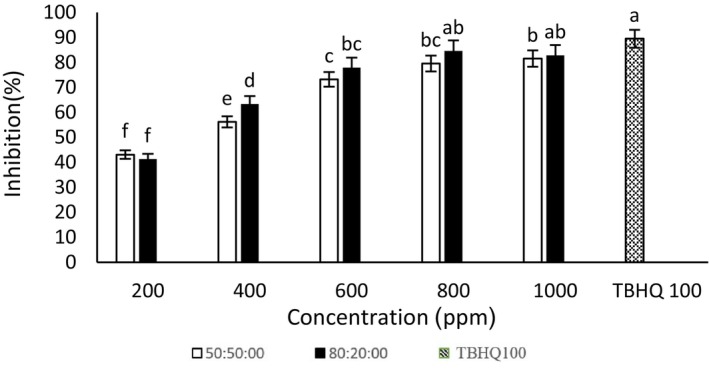
2,2‐diphenyl‐1‐picrylhydrazyl (DPPH) radical inhibition of different concentrations of quinoa–ethanol–water extract (50:50) and (80:20). Different letters indicate significant differences (*p* < .05).

According to Figures 1 and 2, the concentrations of 800 and 1000 μg/mL ethanol–water extract (80:20) ginseng extract showed an insignificant difference in the inhibitory percentage. As a result, 800 μg/mL was introduced as the best concentration of the radical scavenging activity of ethanol–water (80:20) of ginseng extract. The concentration of 800 μg/mL ethanol–water extract (50:50) of ginseng was also considered the best concentration. Also, there was an insignificant difference in the radical inhibition percentage between the concentrations of 800 and 1000 μg/mL ethanol–water extract (50:50) quinoa extract. The concentration of 800 μg/mL was considered the best concentration of this extract. The concentrations of 800 and 1000 μg/mL ethanol–water (80:20) quinoa extract showed an insignificant difference. The best concentration (82% of radical inhibition) of ethanol–water extract (80:20) of quinoa concentration was 800 μg/mL. In this study, the quinoa extract had a better DPPH radical inhibitory effect than the ginseng extract. Because of the ethanol–water extract (80:20) of quinoa at a concentration of 800 μg/mL, 82%, and the ethanol–water extract (80:20) of ginseng at a concentration of 800 μg/mL, 78% showed a radical inhibitory effect. Bravi et al. (2024) stated that quinoa powder showed 75.77 TE/g (Trolox equivalent per gram) in the DPPH assays (Bravi et al., 2024). In the current study, TBHQ had a higher radical scavenging activity than ginseng extracts, which is consistent with the results of Chaari et al. (2018). Also, with the concentration of 800 and 1000 μg/mL of ethanol–water extract (80:20) of quinoa, there was an insignificant difference in the amount of DPPH radical inhibition, which was in the same direction as the research results of Jafari et al. (2022). According to the results, with the increase in the concentration of ginseng and quinoa extracts, the DPPH radical inhibition effect increases, which was consistent with the research of Kamkar (2009). The DPPH free radical inhibition activity in plant extracts shows the ability of extracts to donate hydrogen. The presence of phenolic compounds increases the DPPH free radical inhibition effects (Leong & Shui, 2002).

#### 3.3.2 β‐Carotene: linoleic acid bleaching assay

**FIGURE 3:**
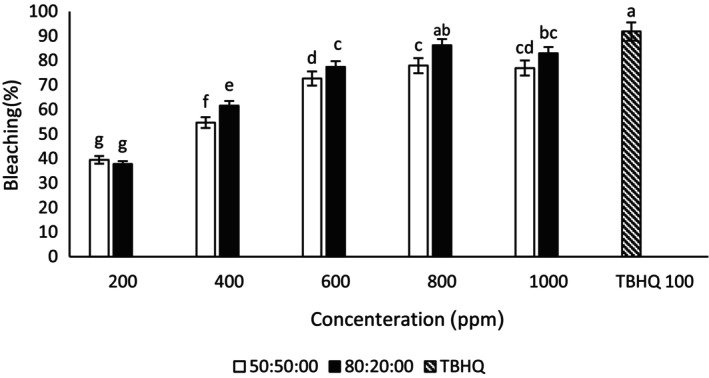
Bleaching of β‐carotene: linoleic acid of different concentrations of ginseng–ethanol–water extract (50:50) and (80:20). Different letters indicate significant differences (*p* < .05).

**FIGURE 4:**
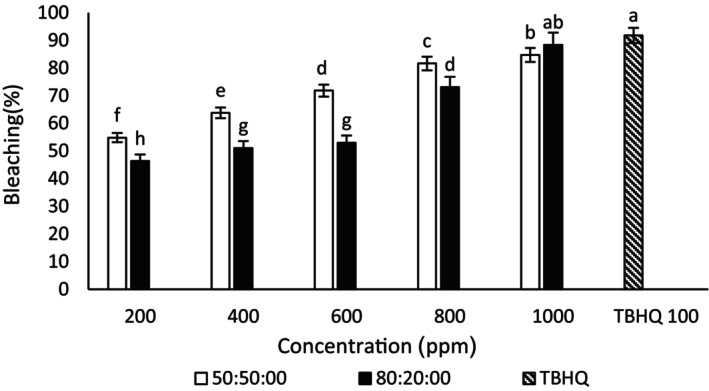
Bleaching of β‐carotene: linoleic acid of different concentrations of quinoa–ethanol–water extract (50:50) and (80:20). Different letters indicate significant differences (*p* < .05).

In the current study, the concentrations of 600, 800, and 1000 μg/mL ethanol–water ginseng extract (50:50) had no significant difference (Figure 3). As a result, 600 μg/mL was introduced as the best concentration of ethanol–water ginseng extract (50:50) in this assay. Also, the concentrations of 800 and 1000 μg/mL ethanol–water extract of ginseng (80:20) did not have statistically significant differences. The concentration of 800 μg/mL was considered the best ethanol–water concentration of ginseng (80:20). A similar result was seen in the ethanol–water extract of quinoa (50:50) (Figure 4); 800 μg/mL was the best concentration of quinoa–ethanol–water extract (50:50) in this assay. The concentration of 1000 μg/mL ethanol–water quinoa extract (80:20) also had the highest antioxidant effect among other concentrations of this extract. In this test, ginseng extract performed better than quinoa extract. The concentration of 800 μg/mL ethanol–water–ginseng extract (80:20) showed no significant difference with TBHQ in this test. In this respect, it competed with TBHQ. Also, no significant difference was observed in the antioxidant activity of TBHQ and 1000 μg/mL concentration of ethanol–water extract (80:20) of quinoa. But it had higher antioxidant activity than other extracts, which was in line with the results of Jafari et al. (2022). In the β‐carotene: linoleic acid bleaching assay, increasing the concentration of ginseng and quinoa extracts increased the amount of antioxidant activity, and it was in the same direction as the results of Bamdad et al. (2006). Mansor et al. (2022) stated that phenolic compounds in ginseng root have high antioxidant capacity (Mansor et al., 2022). Research has shown that direct relationship between the extract antioxidant powers and the prevention of β‐carotene color reduction (Bamdad et al., 2006).

#### 3.3.3 Ferric reducing antioxidant power assays

**FIGURE 5:**
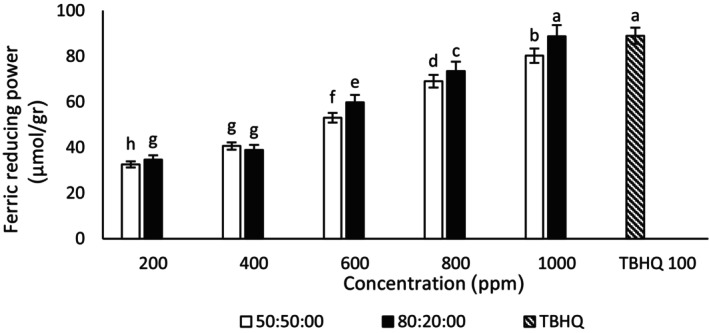
Ferric reducing antioxidant power of different concentrations of ginseng–ethanol–water extract (50:50) and (80:20). Different letters indicate significant differences (*p* < .05).

**FIGURE 6:**
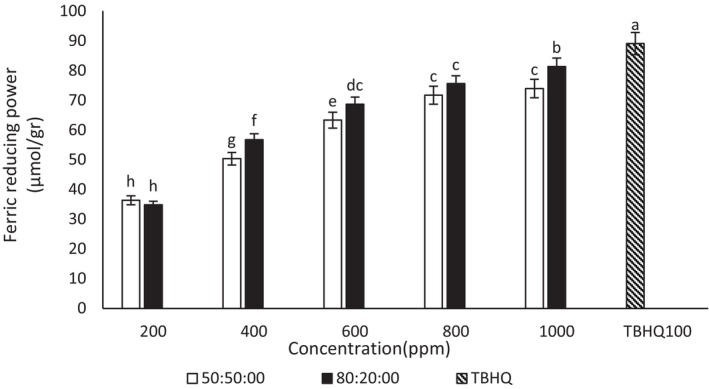
Ferric reducing antioxidant power of different concentrations of quinoa–ethanol–water extract (50:50) and (80:20). Different letters indicate significant differences (*p* < .05).

The results showed with the increase in the concentration of ginseng and quinoa extracts, the FRAP of the extracts increased (Figures 5 and 6). In the current study, the highest FRAP was observed in the concentration of 1000 μg/mL ginseng–ethanol–water extract (80:20) and (50:50). Also, there was an insignificant difference in FRAP between the concentrations of 800 and 1000 μg/mL ethanol–water (50:50) quinoa extract. In this test, ginseng extract performed better than quinoa extract. The FRAP of 1000 μg/mL concentration of ethanol–water ginseng extract (80:20) was not significantly different from the FRAP of TBHQ. Also, the FRAP of TBHQ was higher than all concentrations of quinoa–ethanol–water extract (50:50). A significant difference was observed between different concentrations of the extract. Bravi et al. (2024) stated that quinoa powder showed 3.58 TE/g in FRAP assays (Bravi et al., 2024). The reductive property depends on the electron‐donating ability of the compounds. By increasing the number of phenolic compounds in the extract, FRAP of the extract increases (Zhang et al., 2019). This result is consistent with another study that observed that FRAP increased with the increase in extract concentration (Saini et al., 2020). A study is consistent with the results obtained from this test (Jafari et al., 2022).

In the tests to determine the antioxidant activity of the extract, it was observed that the ethanol–water extracts (80:20) had better antioxidant activity than the ethanol–water extracts (50:50). Also, the concentration of 800 μg/mL extracts had high antioxidant activity. Therefore, it was used for nanoencapsulation.

### 3.4 Characteristics of nanoencapsulates

The highest viscosity was observed in the nanoemulsion containing ginseng + quinoa extract with a chitosan and chitosan/whey protein wall (Table 5). The lowest viscosity was observed in the nanoemulsion containing extract with a whey protein wall. An insignificant difference was observed between the viscosity of nanoemulsions containing ginseng + quinoa extract and quinoa extract with the chitosan wall and the whey protein/chitosan wall and a nanoemulsion containing the ginseng extract with the chitosan wall. Also, an insignificant difference was observed between the viscosity of nanoemulsion containing ginseng extract with chitosan and whey protein/chitosan walls. Each nanoemulsion containing extract with the chitosan wall and whey protein/chitosan wall had an insignificant difference. They had a higher viscosity than the nanoemulsion containing the same extract with a whey protein wall. Mohammadi et al. (2016) stated that the viscosity of emulsions stabilized with two walls (whey protein/pectin) is higher than the viscosity of emulsions stabilized with whey protein alone (Mohammadi et al., 2016). Research has shown that higher viscosity of wall materials reduces particle size, increases EE, and increases emulsion stability, which are consistent with our results (Jafari et al., 2022). Higher viscosity indicates greater resistance to particle movement, avoiding coalescence and resulting in smaller particles. Of course, a wall may have a higher viscosity and particle size is larger, which expresses that particle size is not only affected by the viscosity of the emulsion but also by the inherent emulsifying properties of each type of material (Carneiro et al., 2013).

**TABLE 5:** Properties of nanoemulsions.

Nanoemulsions type	Wall type	Viscosity (mPa/s)	Particle size (nm)	Polydispersity index	Zeta potential (mv)	Encapsulation efficiency (%)
Nanoemulsions of the extracts in a ratio of 1:1	Chitosan	5.30 ± 0.08^a^	275.47 ± 12.26^d^	0.31 ± 0.005^b^	−30.8 ± 1.67^ab^	68.03 ± 1.75^c^
	Whey protein	3.73 ± 0.06^c^	387.13 ± 10.93^a^	0.25 ± 0.005^f^	−31 ± 1.06^ab^	59.9 ± 0.94^g^
	Whey protein/Chitosan	5.30 ± 0.21^a^	330.17 ± 5.48^b^	0.28 ± 0.005^d^	−32.6 ± 0.58^c^	64.43 ± 1.30^fd^
	Chitosan	5.08 ± 0.07^ab^	250.67 ± 11.74^e^	0.34 ± 0.006^a^	−29.56 ± 1.60^ab^	72.79 ± 1.87^a^
Quinoa extract	Whey protein	3.58 ± 0.06^c^	347/91 ± 11.53^b^	0.27 ± 0.005^de^	−29.92 ± 0.82^ab^	64.49 ± 0.55^fd^
Nanoemulsions	Whey protein/Chitosan	5.09 ± 0.20^ab^	303.75 ± 4.98^c^	0.30 ± 0.005^bc^	−31.29 ± 0.56^ab^	68.94 ± 1.39^bc^
	Chitosan	4.99 ± 0.07^ab^	269.67 ± 11.24^de^	0.33 ± 0.006^a^	−30.46 ± 1.22^a^	71.13 ± 1.59^ab^
Ginseng extract	Whey protein	3.60 ± 0.13^c^	379.17 ± 16.15^a^	0.26 ± 0.005^e^	−29.23 ± 1.28^a^	63.20 ± 0.54^f^
Nanoemulsions	Whey protein/Chitosan	4.95 ± 0.22^b^	331.24 ± 1.19^b^	0.29 ± 0.005^c^	−30.61 ± 1.04^ab^	66.75 ± 0.42^cd^

*Note*: Different letters in a column indicate significant differences (*p* < .05).

According to the obtained results, the nanoencapsulate with a chitosan wall had the smallest particle size, and the nanoencapsulate with a whey protein wall had the largest particle size. Among the prepared nanoencapsulates, nanoencapsulates containing quinoa extract with chitosan wall had the smallest particle size, and nanoencapsulates containing ginseng + quinoa extract with whey protein wall had the largest nanoencapsulate size (Table 5). Da Silva et al. (2021) stated in a research that particles nanoencapsulated by chitosan alone have a smaller size, which is consistent with the results of this research (Da Silva et al., 2021). In general, the smaller the size of the particles, the higher their stability in the emulsion due to their higher resistance to the force of gravity due to their upward movement. They also have more suitable functional characteristics (Fathiazad & Hamedeyazdan, 2011). In this research, the size of nanoencapsulates varied from 387.13 to 250.67 nm. In this research, the size of nanoencapsulates varied from 250.67 to 387.13 nm and showed a statistically significant difference. The type of wall material has an effect on the size of the capsules, which can be considered as the reason for the slight difference in the obtained sizes (Razavizadeh et al., 2015).

The PDI indicates particle dispersion. In the current study, the PDI in different samples varied between 0.25 and 0.34. There was a significant difference between them. The lowest PDI was observed in the nanoemulsion containing a mixed extract of ginseng and quinoa with a whey protein wall. The highest PDI was assigned to nanoemulsion containing quinoa extract with a chitosan wall. According to the obtained data (Table 5), nanoencapsulates containing quinoa extract (whey protein wall) and nanoencapsulates containing ginseng and ginseng + quinoa extract (whey protein and whey protein/chitosan walls) had a PDI of less than 0.3, which showed that the size distribution was homogeneous. Yadollahi et al. (2023) reported the average particle size and PDI of nanoencapsulated composites by chitosan/whey protein to be 248.5 nm and 0.41, respectively (Yadollahi et al., 2023). The PDI of less than 0.3 indicates a narrow particle size distribution and favorable uniformity in nanoencapsulates, which expresses a homogeneous system (Lee et al., 2019).

Zeta potential expresses the magnitude of electric charge and electrostatic reactions between particles in suspension. In this research, the zeta potential was negative in all the examined samples (Table 5). The lowest and highest zeta potentials were observed in nanoemulsion containing ginseng + quinoa and ginseng extract with whey protein wall as −32.6 and −29.23 mv, respectively. Nanoemulsions containing ginseng + quinoa (1:1) with chitosan/whey protein powder wall showed the most zeta potential (−32.6 mv). Da Silva et al. (2021) stated in a research that zeta potential of chitosan nanoparticles is shown to be +28.0 (Da Silva et al., 2021). The higher value of the zeta potential means the presence of more repulsive forces between the droplets and their less tendency to stick together. In this case, the emulsion droplets repel each other and lead to the stability of the system (Freitas & Müller, 1998). Nanoemulsions with zeta potentials greater than +30 mV or less than −30 mV are expected to be very stable because the droplets are sufficiently charged to enable interparticle repulsive forces to overcome them (Salvia‐Trujillo et al., 2013).

In this research, among the three types of wall materials examined, the highest EE was related to the nanoencapsulate with the chitosan wall (Table 5). The lowest EE was related to the nanoencapsulate with a whey protein wall. According to the information obtained from this research, the highest and lowest EE values were assigned to nanoencapsulates containing quinoa extract with chitosan wall (72.79%) and the nanoencapsulates containing ginseng+quinoa extract with whey protein wall (59.9%), respectively. Also, a statistically significant difference was observed between different treatments. According to the current study, in the research of Esquerdo et al. (2019), chitosan encapsulates showed more efficiency than encapsulates with chitosan/gelatin wall (10:90) and (30:70). In their research, microencapsulates with chitosan/gelatin wall (30:70) had lower efficiency than microencapsulates with chitosan/gelatin wall (10:90). The difference caused by increasing the amount of gelatin showed that gelatin is ineffective as a polymer coating. The results showed that the coating quality of oil droplets was directly affected by the size of the polymer chain (protein and polysaccharide). Smaller molecules may not form a powerful system to protect the nucleus (Esquerdo et al., 2019). These results show the effect of the type of material used in the wall on the EE. Da Silva et al. (2021) stated that the highest EE of phenolic compounds by chitosan was 52% (Da Silva et al., 2021). In the research of Duan et al. (2011), EE of whey protein/chitosan films was 54% to 66%, which was close to the results of our research (Duan et al., 2011).

### 3.5 Scanning electron microscopy image of nanoencapsulates

**FIGURE 7:**
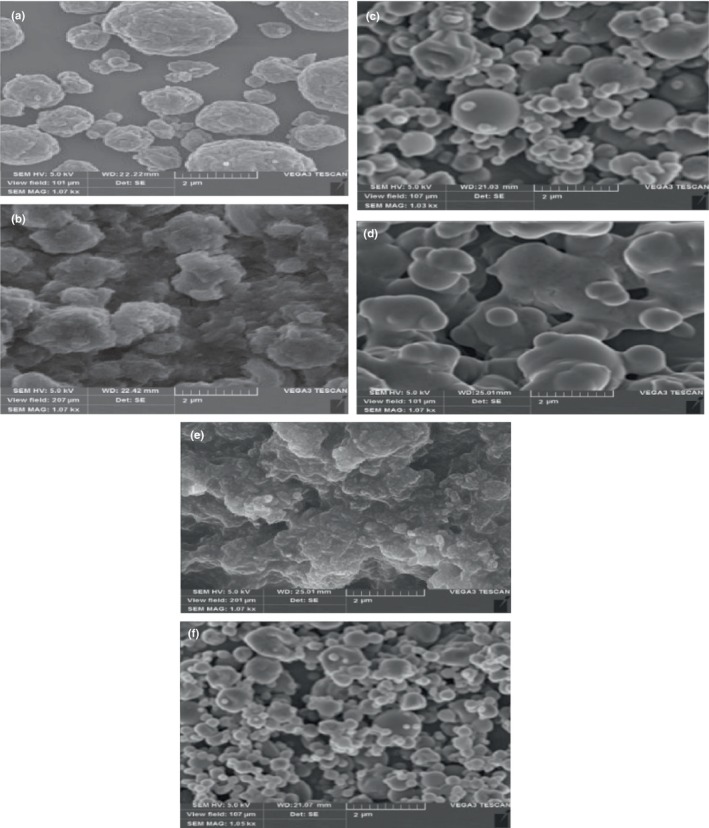
Scanning electron microscopy images of nanoencapsulates containing optimal ginseng and quinoa extracts with whey protein, chitosan wall, and their combination. (a) ginseng–whey protein; (b) ginseng–chitosan; (c) ginseng–whey protein/chitosan; (d) quinoa–whey protein; (e) quinoa–chitosan; (f) quinoa–whey protein/chitosan.

One of the most important factors in encapsulating extracts is the surface morphology of the capsules. Examining the surface morphology of the nanoencapsulates showed that the nanoencapsulates prepared from the combined coating had a more uniform and smooth structure compared to the chitosan wall and the whey protein wall alone (Figure 7). Yadollahi et al. (2023) stated that the presence of whey protein caused smoothness and sphericity of the nanoencapsulated particles. Chitosan caused nonuniformity of the nanoencapsulated particles (Yadollahi et al., 2023). It indicates that the combined wells are more suitable for encapsulation. Spherical structures without cracks have less permeability to gases, oxygen, and unpleasant oil leakage to the particle surface (Drusch et al., 2007). Also, emulsion drying rate, wall material composition, and capsule production conditions can affect the surface characteristics of capsules. For example, if the emulsion drying temperature is high, it loses moisture faster. As a result, the encapsulates become more spherical, and the EE increases (Kenari et al., 2020; Nijdam & Langrish, 2006).

### 3.6 Choosing the best type of nanoencapsulate

A general comparison of the characteristics of prepared nanoencapsulates showed that the highest EE and the smallest particle size (with the most important determining factors in an effective encapsulation) are related to nanoencapsulates with chitosan walls. Among the nanoencapsulated extracts in the chitosan wall, the quinoa extract with the highest EE and the smallest particle size was recognized as the best capsule. The nanoencapsulates containing ginseng and ginseng + quinoa extracts were placed after the nanoencapsulates containing quinoa extract with a slight difference. Therefore, these three types of nanoencapsulates containing ethanol–water extract (80:20) ginseng, quinoa, and ginseng + quinoa (concentration 800 μg/mL) were used in PV, TBA value, and release rate of phenolic compounds of the extract.

### 3.7 Peroxide value

**FIGURE 8:**
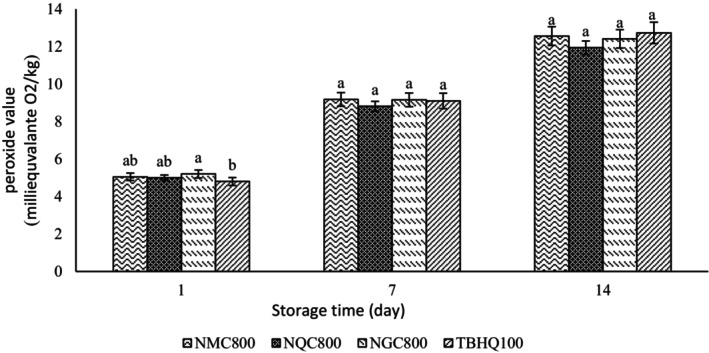
Changes in peroxide value in treatments containing extract nanoencapsulated in chitosan wall and synthetic antioxidant TBHQ. NMC800, Mixture of quinoa and ginseng extract with chitosan wall (800 ppm); NQC800, Quinoa extract with chitosan wall (800 ppm); and NGC800, Ginseng extract with chitosan wall (800 ppm). Different letters indicate significant differences (*p* < .05).

Primary oxidation products were measured by PV. In this study, PV changes of treatments containing extracts were investigated over 14 days and compared with that of the treatment containing TBHQ (Figure 8). The treatment containing quinoa extract in the chitosan wall had the lowest PV, so it was recognized as the best treatment. After that, the treatment containing ginseng extract had the lowest PV in the chitosan wall. Also, an insignificant difference was observed in the PV in different samples. The high level of PV indicates the lower oxidative stability created in the treatments tested in this research. It is consistent with the results of Mansour and Sindi (2024). Perhaps, the reason for the higher oxidative stability created in the treatment containing nanoencapsulated quinoa extract in the chitosan wall compared to the other treatments can be related to the type of phenolic compounds currently used in this extract, which was able to create a higher synergistic effect with the wall material than other treatments. PV in all samples increased over time, which was in the same direction as the results of Rashidaie Abandansarie et al. (2019). PV in the treatment containing TBHQ on the 14th day was slightly higher than the other treatments, which was in line with the results of Yang et al. (2016). In general, extracts prevent fat oxidation due to the presence of phenolic compounds. Especially, their antioxidant effect is higher if they are encapsulated, which indicates the ability of the wall to improve the antioxidant activity and increase the availability of the extract (Javadian et al., 2017). Chitosan acts as an antioxidant by eliminating oxygen radicals such as hydroxyl, alkyl, and superoxide and improves the antioxidant activity of the extract (Younes & Rinaudo, 2015).

### 3.8 Thiobarbituric acid value

**FIGURE 9:**
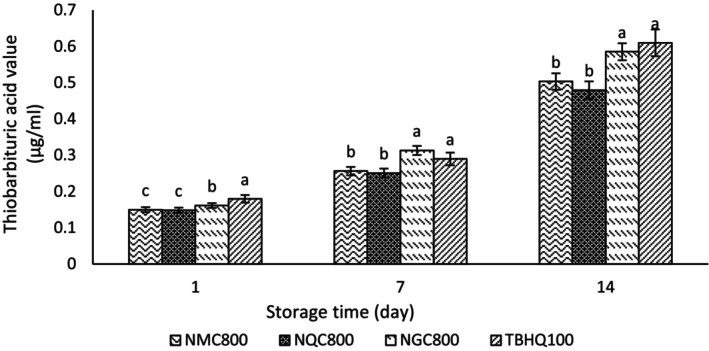
Changes in thiobarbituric acid value in treatments containing extract nanoencapsulated in chitosan wall and synthetic antioxidant TBHQ. NMC800, Mixture of quinoa and ginseng extract with chitosan wall (800 ppm); NQC800, Quinoa extract with chitosan wall (800 ppm); and NGC800, Ginseng extract with chitosan wall (800 ppm). Different letters indicate significant differences (*p* < .05).

The TBA value was used to determine secondary oxidation products. In the current study, the TBA value was low in all samples in the first few days (Figure 9). Over time, due to the decomposition of the primary oxidation products, the TBA value increased, which indicates the increasing process of oxidation in the dairy cream and the creation of secondary oxidation products such as ketones and aldehydes during the storage period. In the current study, an insignificant difference was observed in TBA value in the treatment containing TBHQ and ginseng extract. The treatments containing ginseng + quinoa extract and quinoa extract showed an insignificant difference in TBA value. The treatment containing TBHQ and ginseng extract showed a significant difference in TBA value compared to the treatments containing ginseng + quinoa extract and quinoa extract. The highest and lowest TBA values in the treatment containing TBHQ and quinoa extract with chitosan wall were observed, respectively. According to obtained results, the treatment containing nanoencapsulated quinoa extract in the chitosan wall with the lowest TBA value was recognized as the best treatment. Also, the chitosan coating of the cinnamon extract led to a decrease in the TBA of rainbow trout fillets at 4°C (Ojagh et al., 2010). According to the research of Rezaei Savadkouhi et al. (2020), in this test, the chitosan wall played an essential role in improving the antioxidant activity of the extract (Rezaei Savadkouhi et al., 2020). In a study, the TBA value in oil containing TBHQ was higher than the amount of TBA in oil containing nanoencapsulated extract (Razavi & Kenari, 2021). In this regard, a study showed TBA value increased during the storage period in the refrigerator due to the oxidation of unsaturated fatty acids (Ojagh et al., 2010). The researchers stated that the addition of date seed extract to butter during storage for 21 days had less TBA value than the control sample. Also, during storage, the TBA value of butter containing extract increased, which is consistent with the results of this study (Mansour & Sindi, 2024).

### 3.9 Release properties

**FIGURE 10:**
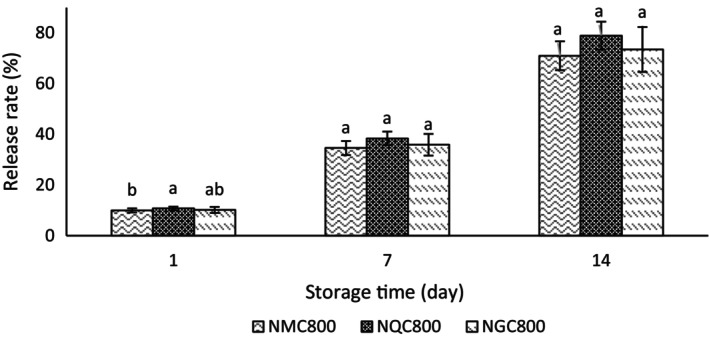
Comparison of the release rate of phenolic compounds in treatments containing extract nanoencapsulated in chitosan wall and synthetic antioxidant TBHQ. NMC800, Mixture of quinoa and ginseng extract with chitosan wall (800 ppm); NQC800, Quinoa extract with chitosan wall (800 ppm); and NGC800, Ginseng extract with chitosan wall (800 ppm). Different letters indicate significant differences (*p* < .05).

According to Figure 10, there was an increase in the release rate of phenolic compounds during the storage period of 1 to 14 days, which is well related to the destruction of the wall material and the release of the extract due to the passage of time. The results of this experiment did not show a significant difference in the release rate of phenolic compounds in different treatments. But in general, the release rate of phenolic compounds in the treatment containing the ginseng+quinoa extract was a little lower than the other two treatments. It is consistent with the research results of Afshari et al. (2023). The biopolymer walls of the capsule swelled by absorbing moisture. At the same time, the glass transition temperature decreased. Finally, the cohesion and interweaving of biopolymer chains decreased. Also, the amount of molecular movement of the encapsulated particles increased. The collapse of the texture of the raw materials and the sudden increase in the molecular coefficient of the effective compounds are influential factors in raising the diffusion coefficient of the compounds from the nanocapsule to the outside and the release rate (Najaf Najafi et al., 2011). Research has shown that the intrinsic properties of the wall material, chemical interactions between the shell material and the encapsulated compound, and the environment affect the release behavior (Adejoro et al., 2019).

## 4 CONCLUSION

Plant extracts with high natural antioxidant properties are suitable substitutes for synthetic antioxidants in dairy products sensitive to oxidation, such as dairy cream. As a result, the nanoemulsion of quinoa and ginseng extracts on the shelf life of dairy cream was investigated. Ginseng extract had the highest TPC and TFC. The most phenolic and flavonoid compounds of ginseng and red quinoa extracts were related to p‐coumaric acid, catechin, ellagic acid, and rutin, respectively. The concentration of 800 ppm of red quinoa extract (ethanol–water solvent (80:20)) in DPPH radical scavenging, bleaching β‐carotene: linoleic acid, and FRAP assays was observed. Nanoemulsions containing ginseng + quinoa (1:1) with chitosan/whey protein powder wall showed the highest viscosity. Nanoemulsions of red quinoa extract with chitosan wall had the smallest particle size, the highest EE, and the PDI. Also, this sample showed the lowest PV and TBA values in dairy cream oil. There was an increase in the release rate of phenolic compounds during the storage period of 1 to 14 days, which is well related to the destruction of the wall material and the release of the extract due to the passage of time. The current study confirmed that plant extracts have a high potential for the presence of dairy products, especially dairy creams, due to their antioxidant properties.

## AUTHOR CONTRIBUTIONS

**Hadis Aziminezhad:** Formal analysis (equal); methodology (equal); software (equal); visualization (equal); writing – original draft (equal). **Reza Esmaeilzadeh Kenari:** Conceptualization (equal); data curation (equal); investigation (equal); project administration (equal); supervision (equal); writing – review and editing (equal). **Zeynab Raftani Amiri:** Investigation (equal); project administration (equal); resources (equal); supervision (equal); writing – review and editing (equal).

## CONFLICT OF INTEREST STATEMENT

Authors declare no conflict of interest.

## Data Availability

Research data are not shared.
